# High prevalence of rheumatoid arthritis and its risk factors among Tibetan highlanders living in Tsarang, Mustang district of Nepal

**DOI:** 10.1186/s40101-022-00283-3

**Published:** 2022-04-02

**Authors:** Hiroaki Arima, Sweta Koirala, Kotaro Nema, Masayuki Nakano, Hiromu Ito, Kapil Madi Poudel, Kishor Pandey, Basu Dev Pandey, Taro Yamamoto

**Affiliations:** 1 Department of International Health and Medical Anthropology, Institute of Tropical Medicine, Nagasaki University, 1-12-4 Sakamoto, Nagasaki, 852-8523 Japan; 2 Nepal Development Society, Bharatpur-5, Chitwan, Nepal; 3 School of Medical Sciences, Nagasaki University, 1-12-4 Sakamoto, Nagasaki, Japan; 4 Department of Nutrition, Faculty of Health Sciences, Kochi Gakuen University, 292-26 Asahitenjin-cho, Kochi, Japan; 5 Institute of Medicine, Tribhuvan University, Maharajgung, 44600 Nepal; 6 Central Department of Zoology, Tribhuvan University, Kirtipur, Nepal; 7 Department of Molecular Epidemiology, Institute of Tropical Medicine, Nagasaki University, 1-12-4 Sakamoto, Nagasaki, Japan; 8 Graduate School of Biomedical Sciences, Nagasaki University, 1-12-4 Sakamoto, Nagasaki, Japan; 9 Leading Program, Graduate School of Biomedical Sciences, Nagasaki University, 1-12-4 Sakamoto, Nagasaki, Japan

**Keywords:** Rheumatoid arthritis, Tibetan highlander, Mustang

## Abstract

**Background:**

In Tsarang (at 3560 m), which is located in Mustang, 62.7% of the residents answered that they had a subjective medical history of arthritis, and 41.1% of the residents answered that their families had a subjective medical history of arthritis on a survey conducted in 2017. The expression of hypoxia-inducible factor (HIF) and its effects are deeply involved in hypoxic adaptation in Tibetan highlanders. At the same time, HIF is also related to the onset of rheumatoid arthritis. Therefore, the adaptive mechanism acquired by Tibetan highlanders may promote the development of rheumatoid arthritis. The prevalence of rheumatoid arthritis is estimated to be approximately 0.5–1.0% worldwide. The objective of this study was to estimate the prevalence of rheumatoid arthritis in Tsarang residents using existing diagnostic criteria and to explore its risk factors.

**Methods:**

An epidemiological survey was conducted in Tsarang in 2019. Data obtained from anthropometry and questionnaires were statistically analyzed. Biochemical measurements using blood samples were also performed, and the results were used to assess arthritis status. Residents’ joint status was scored, and arthritis was assessed based on the clinical disease activity index and ACR/EULAR 2010 criteria.

**Results:**

Twenty-seven males and 50 females participated in this survey. In Tsarang, ACR/EULAR 2010 classified 4.3% of males and 7.1% of females as having rheumatoid arthritis, indicating a very high estimated prevalence. We also performed a multivariate analysis to explore its risk factors, and two factors, older age (standardized parameter estimate = 4.84E−01, 95% CI = [9.19E−02, 8.76E−01], *p* = 0.0170) and a history of living in urban areas (standardized parameter estimate = − 5.49E−01, 95% CI = [− 9.21E−01, 1.77E−01], *p* = 0.0050), significantly contributed to the higher ACR/EULAR 2010 score in females. In addition, three factors, having no spouse (standardized parameter estimate = 3.17E−01, 95% CI = [5.74E−02, 5.77E−01], *p* = 0.0179), having a smoking habit (standardized parameter estimate = 2.88E−01, 95% CI = [1.71E−02, 5.59E−01], *p* = 0.0377), and a history of living in urban areas (standardized parameter estimate = − 3.69E−01, 95% CI = [− 6.83E−01, − 5.60E−02], *p* = 0.0219), resulted in significantly higher clinical disease activity index scores in females. Furthermore, smoking habits were found to significantly increase blood hyaluronic acid in both males (standardized parameter estimate = 6.03E−01, 95% CI = [3.06E−01, 9.01E−01], *p* = 0.0020) and females (standardized parameter estimate = 4.87E−01, 95% CI = [5.63E−02, 9.18E−01], *p* = 0.0291).

**Conclusions:**

In this study, we evaluated the symptoms of arthritis and estimated the prevalence of rheumatoid arthritis using classification criteria for Tibetan highlanders who have adapted to the hypoxic environment and fostered their own culture. The high prevalence of rheumatoid arthritis among Tsarang residents suggests that the hypoxic adaptation mechanism involving HIF in Tibetan highlanders may promote the onset or exacerbation of rheumatoid arthritis. The high prevalence of rheumatoid arthritis among Tibetan highlanders may be related not only to the environmental factors analyzed in this study but also to hypoxic adaptation genes. Further investigation is needed to clarify the genetic factors involved.

## Background

### A medical history of arthritis among Tsarang residents

Archeological studies suggest that humans entered the Tibetan Plateau 50,000 to 25,000 years ago [1, 2]. In addition, especially in Mustang, it is thought that human beings began to live there approximately 4500 years ago [3]. Mustang, which was the focus area for this research, is a mountainous area in the Annapurna Conservation Area, located approximately 206.1 km in a straight line from the capital Kathmandu [4]. Additionally, this district is a region where the Mustang kingdom was founded in 1440. The Tibetan highlanders currently live there. The Kingdom of Mustang survived as an autonomous kingdom until 2008 and is a rare area that has long restricted exchanges with foreign countries [5–7]. Therefore, people who live in Mustang are highly likely to inherit genes increasing adaptation to the hypoxic environment that were acquired by ancient Tibetan highlanders as a population.

While the hypoxic adaptation mechanism of these Tibetan highlanders enables environmental adaptation, it makes them vulnerable to aging and lifestyle changes, and a possibility of promoting the onset of diabetes has been reported [8]. An epidemiological study conducted in Tsarang in 2017 also revealed an increase in hemoglobin concentration with age in only females [9]. In Tsarang village (altitude 3560 m) in Mustang, residents’ health conditions have not been clarified because health surveys by the government have not been thoroughly conducted. In 2017, an epidemiological survey of Tibetan highlanders living in Tsarang was conducted to investigate health status, especially regarding noncommunicable diseases and their risk factors. As a result, in terms of questionnaires related to arthritis, 62.7% of the residents answered that they had a subjective medical history of arthritis, and 41.1% of the residents answered that their families had a subjective medical history of arthritis [10]. From this survey, it became clear that arthritis has become a health problem among the residents of Mustang.

### Prevalence of rheumatoid arthritis and its pathology associated with hypoxia

The prevalence of rheumatoid arthritis is estimated to be approximately 0.5–1.0% worldwide [11–13]. On the other hand, in Southern Europe, including Greece, the prevalence rate is as low as 0.18–0.34% [14–16]. Conversely, the prevalence rate is as high as 5.3% [17] in native American Pima Indians and 6.8% in Chippewa Indians [18]. To date, it has been reported that genetic differences may affect the prevalence of rheumatoid arthritis [19], but differences in the classification criteria used in each survey also heavily contribute to differences in prevalence.

Rheumatoid arthritis is an autoimmune disease, and autoantibodies such as rheumatoid factor and anti-cyclic citrullinated peptide (CCP) antibody are involved in the onset of the disease. In the early stage, synovitis, bilateral joint swelling, and pain occur in the small joints. As the condition progresses, the joints become deformed, the inflamed areas spread to the wrists, knees, elbows, shoulders, etc., and this joint destruction has a great impact on the patient’s daily life [20, 21].

### Relationship between hypoxic adaptation and HIF among Tibetan highlanders

The mutant *endothelial per-ARNT-sim domain protein 1* (*EPAS1*) gene inherited from Denisovans is thought to enable Tibetan highlanders to adapt to hypoxic environments [22]. When people living in lowlands go to a hypoxic environment, HIF2 expressed by the *EPAS1* gene is less likely to be degraded, and a hypoxic response is initiated. As a result, the body tries to adapt to hypoxia by increasing the amount of erythropoietin secretion and the concentration of hemoglobin in the blood [23]. On the other hand, many Tibetan high highlanders have mutations specific to the *EPAS1* gene, and having this mutation increases the ability to increase blood NO levels and dilate blood vessels. It is also known that even in a hypoxic environment, the hemoglobin concentration is maintained at the same level as that of people in the lowlands, and the increase in hemoglobin concentration is suppressed. In other words, it is thought that the hypoxic adaptation of Tibetan highlanders improves oxygen circulation by enhancing the vasodilatory effect instead of suppressing the increase in blood hemoglobin concentration [24]. *EPAS1* is the gene that expresses HIF-2α, which is also deeply involved in the pathophysiology of rheumatoid arthritis [25]. We suspect that the hypoxic adaptation mechanism acquired by Tibetan highlanders not only enables environmental adaptation but is also involved in promoting the onset of rheumatoid arthritis.

### Hypothetical risk factors and objectives of this study

It has been reported that age, sex, smoking habits, etc. are risk factors for rheumatoid arthritis [26]. In addition, it has been reported that married people have a longer life expectancy than single people, suggesting that marriage also has some effect on health [27, 28]. Furthermore, when Tibetan highlanders come and go in urban areas (normal oxygen environment), the adaptive mechanism may be switched on and off, which may add excessive stimulation to HIF expression. We thought that such changes in life and irritation to the body, such as drinking habits in a hypoxic environment, may be risk factors for rheumatoid arthritis through disturbance of HIF expression.

We suspect that the hypoxic adaptation mechanism acquired by Tibetan highlanders not only enables environmental adaptation but is also involved in promoting the onset of rheumatoid arthritis. Therefore, in this study, to clarify the actual situation of rheumatoid arthritis, to estimate the prevalence of rheumatoid arthritis, and to explore its risk factors among Tibetan highlanders, we conducted an epidemiological survey in Tsarang.

## Methods

### Study population

In June 2019, we conducted a cross-sectional study aimed at assessing arthritis status in Tibetan highlanders living in the village of Tsarang in the Mustang district of Nepal. The population of Tsarang is 452 people (217 males and 235 females), and among them, 179 males and 190 females are over the age of 18 years [29]. A venue was set up at the Health Post in Tsarang Village to conduct a survey. Children under the age of 18 years, pregnant females, patients with diseases such as cancer, and residents not born in the highlands were excluded. In this study, we hypothesize that Tibetan highlanders who have genetically acquired hypoxic adaptation over many years may be more likely to develop rheumatoid arthritis if they continue to be exposed to the hypoxic environment from when they were born. We planned the investigation based on this hypothesis. Therefore, we set such exclusion criteria to exclude nonhypoxic-adapted residents who were born and raised in the lowlands and migrated to the highlands.

### Questionnaire survey

In the questionnaire survey, we investigated the basic attributes of the subjects, the consumption of cigarettes and alcohol, and the status of outpatient medication and dysfunction due to arthritis. In addition, to calculate the score of the clinical disease activity index (CDAI), we collected data on joint symptoms such as the number and location of joints with swelling and tenderness, the degree of pain, bilateral disability, and the duration of joint pain [30]. The CDAI is an index that was originally used for evaluating the activity of patients diagnosed with rheumatoid arthritis, but in this study, it was used to evaluate the degree of joint damage. The score is calculated according to the number of tender joints, the number of swollen joints, the subject’s visual analog scale (VAS), and the doctor’s VAS. Interviews using these questionnaires were conducted by Nepali- and Tibetan-speaking doctors and nurses.

### Anthropometric and biochemical measurements

Trained researchers and medical staff measured the subject’s height, weight, blood pressure, hemoglobin concentration, and saturation of peripheral oxygen (SpO_2_). A tape measure was attached to the wall to measure height, and the Health meter HA-851-BL (TANITA, Tokyo, Japan) was used to measure weight. An automatic sphygmomanometer (OMRON Model, HEM-7210, Kyoto, Japan) was used to measure blood pressure, an ASTRIM FIT health monitoring analyzer (Sysmex, Kobe, Japan) was used to measure hemoglobin concentration, and a pulse oximeter (Masimo Radical V 5.0, Masimo Corp, CA, USA) was used to measure SpO_2_. Body mass index (BMI) was calculated from height and weight (BMI = Weight (kg)/Height (m)^2^). The collected blood samples were centrifuged at 3000 rpm in Tsarang, and plasma was separated. Blood samples were transported to the Institute of Tropical Medicine, Nagasaki University according to the United Nations standards with UN3373 (Category B) containers, and the levels of rheumatoid factor (RF), anti-CCP antibody, C-reactive protein (CRP), and hyaluronic acid were measured by the outsourcing examination company (SRL, Inc. Tokyo, Japan).

The positive criteria for RF and anti-CCP antibody were > 15 IU/mL and ≥ 4.5 U/mL, respectively, and the high value criteria for CRP and hyaluronic acid were > 0.3 mg/dL and ≥ 50.0 ng/mL, respectively. In addition, for RF and anti-CCP antibody, more than 3 times the standard was considered very high. Furthermore, rheumatoid arthritis was classified according to the ACR/EULAR 2010 classification criteria based on biochemical measurement results, the degree of joint damage, and the duration of arthritis [31].

Although there are no major fluctuations, the cutoff values to be set vary by country and facility [32, 33]. In fact, the international classification standard ACR/EULAR2010 used in this study stipulates that “the positive standard exceeds the normal value for each facility” [34]. It also stipulates that “high positive is more than 3 times the upper limit of normal value.” Therefore, in this study, classification was performed according to the SRL criteria that were serologically tested.

### Statistical analysis

Data were analyzed by Fisher’s exact tests, Welch’s *t* tests, and Cochrane-Armitage tests for comparison of the rates of each variable and shown as the mean ± standard deviation (SD) or percentage (%). In addition, to explore factors related to the increase in CDAI score and ACR/EULAR2010 score, we performed Spearman’s rank correlation coefficient analysis, linear regression analysis, and multivariate analysis using sociological variables and tobacco/alcohol consumption status as explanatory variables for each objective variable.

Sociological variables included marital status (married or unmarried), alcohol consumption habits (no drinking experience, past drinking history, drinking less than once a week, or drinking more than once a week), smoking habits (no smoking experience, past smoking history, smoking less than once a week, or smoking more than once a week), and living in an urban area (never or past experience living in Kathmandu or Pokhara). Kathmandu (altitude: 1400 m) is the capital city, and Pokhara (altitude: 822 m) is the second largest city in Nepal. Finally, to explore the association between smoking and the development of arthritis, Spearman’s rank correlation coefficient, linear regression analysis, and multivariate analysis were conducted using the data about daily activity restriction, small joint disorders, large joint disorders, or the values for serological factors. In addition, considering that sex is one of the risk factors for rheumatoid arthritis, all analyses were performed by sex in this study. The significance level was set to a *p* value of less than 0.05. R (version 3.5.3), and R studio was used for these statistical analyses.

## Results

### Subjects’ sociological and physiological characteristics

Twenty-seven males and 50 females participated in the field survey at Tsarang. In this study, we set exclusion criteria for subjects, but not all participants were applied to the exclusion criteria. Of the residents who participated, 23 males and 42 females agreed to donate blood, and blood tests were also conducted using blood samples for a total of 65 people. Subjects were classified according to basic attributes and cigarette and alcohol intake, and the numbers for each item and proportions are summarized in Table 1. The ages of males and females were 53.5 ± 11.3 and 51.2 ± 11.8 years, respectively, and there was no significant difference (*p* = 0.3981). Regarding education history, we classified the subjects into 4 groups (no educational history, elementary school, junior high school, high school or more), and the proportion of males and females in each group was denoted. Although a significant sex difference in the four groups of educational history was not identified (*p* = 0.0507), the proportion of non-educated subjects was higher among females (86.0%) than among males (59.3%). The employment rate (male: 25.9%, female: 12.0%, *p* = 0.2004), marital rate (male: 92.6%, female: 86.0%, *p* = 0.4814), and single marriage rate (not polygamy, male: 92.6%, female: 82.0%, *p* = 1.0000) were higher in males than in females. However, there were no statistically significant sex differences. In addition, 85.2% of males and 86.0% of females had experience working in cities (Kathmandu and Pokhara), and there was no significant sex difference (*p* = 1.0000). The prevalence of alcohol consumption was 81.5% for males and 58.0% for females, which was a significant sex difference (*p* = 0.0015), and smoking history was 66.7% for males and 60.0% for females, with no significant sex difference (*p* = 0.6281).

**Table 1:** Demographic and lifestyle characteristics of participants

Variables	Male (*n* = 27)		Female (*n* = 50)		*p* value
	*n*	%	*n*	%	
Age (mean ± SD)	53.5 ± 11.3		51.2 ± 11.8		0.3981
Education					
No formal schooling	16	59.3	43	86.0	0.0507
Less than primary school	6	22.2	3	6.0	
Less than secondary school	4	14.8	2	4.0	
Higher education	1	3.7	2	4.0	
Occupation					
Employed	7	25.9	6	12.0	0.2004
Unemployed	20	74.1	44	88.0	
Current marital status					
Married	25	92.6	43	86.0	0.4814
Not Married	2	7.4	7	14.0	
Marriage form^a^					
Single marriage	25	92.6	41	82.0	1.0000
Polyandry	1	3.7	2	4.0	
Experience living or working at urban					
Yes	23	85.2	43	86.0	1.0000
No	4	14.8	7	14.0	
Alcohol consumption					
Never drinker	5	18.5	29	58.0	0.0015
Drinker	22	81.5	21	42.0	
Smoking					
Never smoker	18	66.7	30	60.0	0.6281
Smoker	9	33.3	20	40.0	

Data were analyzed by Fisher’s exact tests, Welch’s *t* tests, or Cochran-Armitage trend tests for comparison of variables between males and females

Position: After the first paragraph of section “Subjects’ sociological and physiological characteristics” in the “Results” section

^a^One male participant and 7 female participants answered “I do not know” or refused to answer

Table 2 summarizes the physiological values of the subjects. The mean systolic blood pressure was 135.7 ± 26.2 mmHg for males and 116.4 ± 22.6 mmHg for females, which was a significant sex difference (*p* = 0.0023). However, for other values, such as BMI (male: 23.8 ± 3.1, female: 23.7 ± 4.2, *p* = 0.9017), diastolic blood pressure (male: 84.6 ± 13.4 mmHg, female: 78.3 ± 13.1 mmHg, *p* = 0.0517), hemoglobin (male: 13.9 ± 1.9 g/dL, female: 14.4 ± 2.2 g/dL, *p* = 0.3839), and SpO_2_ (male: 89.2 ± 3.2%, female: 88.6 ± 5.1%, *p* = 0.5616), there were no significant sex differences.

**Table 2:** Anthropometric and biochemical characteristics by sex

Variables	Male (*n* = 27)	Female (*n* = 50)	*p* value
	Mean ± SD	Mean ± SD	
Age (years)	53.5 ± 11.3	51.2 ± 11.8	0.3981
BMI (kg/m^2^)	23.8 ± 3.1	23.7 ± 4.2	0.9017
SBP (mmHg)	135.7 ± 26.2	116.4 ± 22.6	0.0023
DBP (mmHg)	84.6 ± 13.4	78.3 ± 13.1	0.0517
Hemoglobin (g/dL)	13.9 ± 1.9	14.4 ± 2.2	0.3839
SpO_2 (%)_	89.2 ± 3.2	88.6 ± 5.1	0.5616

Data were analyzed by Welch’s *t* tests for comparison of variables between males and females

a. Data from 1 female participant did not include BMI, SBP, DBP, or SpO_2_

b. Hemoglobin could not be measured for 5 male participants and 2 female participants due to severe deformation of finger joints or machine troubles

### The effects of arthritis on daily life, use of medications, and hospital visits

The proportion of subjects who felt that arthritis interfered with their daily lives was 68.0% among females and 44.4% among males, but no statistically significant difference was observed (*p* = 0.0543) (Table 3). Regarding the movement of their own joints, 22.2% of males and 26.0% of females answered that they were not satisfied, and there was no significant sex difference (*p* = 0.6825). The number of days of awareness of joint pain in the previous 7 days was 6–7 days in 34.0% of females and 25.9% of males and 0–2 days in 42.0% of females and 59.3% of males. No significant sex difference was found (*p* = 0.2248). Both the outpatient rate due to arthritis symptoms (male: 11.1%, female: 38.0%, *p* = 0.0163) and the medication rate (male: 11.1%, female: 34.0%, *p* = 0.0324) were higher in females.

**Table 3:** Current situation of joints and arthritis among residents

Variables	Male (*n* = 27)		Female (*n* = 50)		*p* value
	*n*	%	*n*	%	
Limitation in usual activity					
Yes	12	44.4	34	68.0	0.0543
No	15	55.6	16	32.0	
Satisfaction with current ability					
Satisfied	13	48.1	22	44.0	0.6825
Neither satisfied nor dissatisfied	8	29.6	15	30.0	
Dissatisfied	6	22.2	13	26.0	
Days with pain in past 7 days					
0–2 days	16	59.3	21	42.0	0.2248
3–5 days	4	14.8	12	24.0	
6–7 days	7	25.9	17	34.0	
Hospitalized for joint symptoms^a^					
Yes	3	11.1	19	38.0	0.0163
No	24	88.9	30	60.0	
Medication for joints					
Yes	3	11.1	17	34.0	0.0324
No	24	88.9	33	66.0	

Data were analyzed with Fisher’s exact tests or Cochran-Armitage trend tests for comparison of variables between males and females

Position: After the first paragraph of section “The effects of arthritis on daily life, use of medications, and hospital visits” in the “Results” section

^a^One female participant did not answer this question

### Evaluation of arthritis by joint damage site and CDAI

**Fig. 1:**
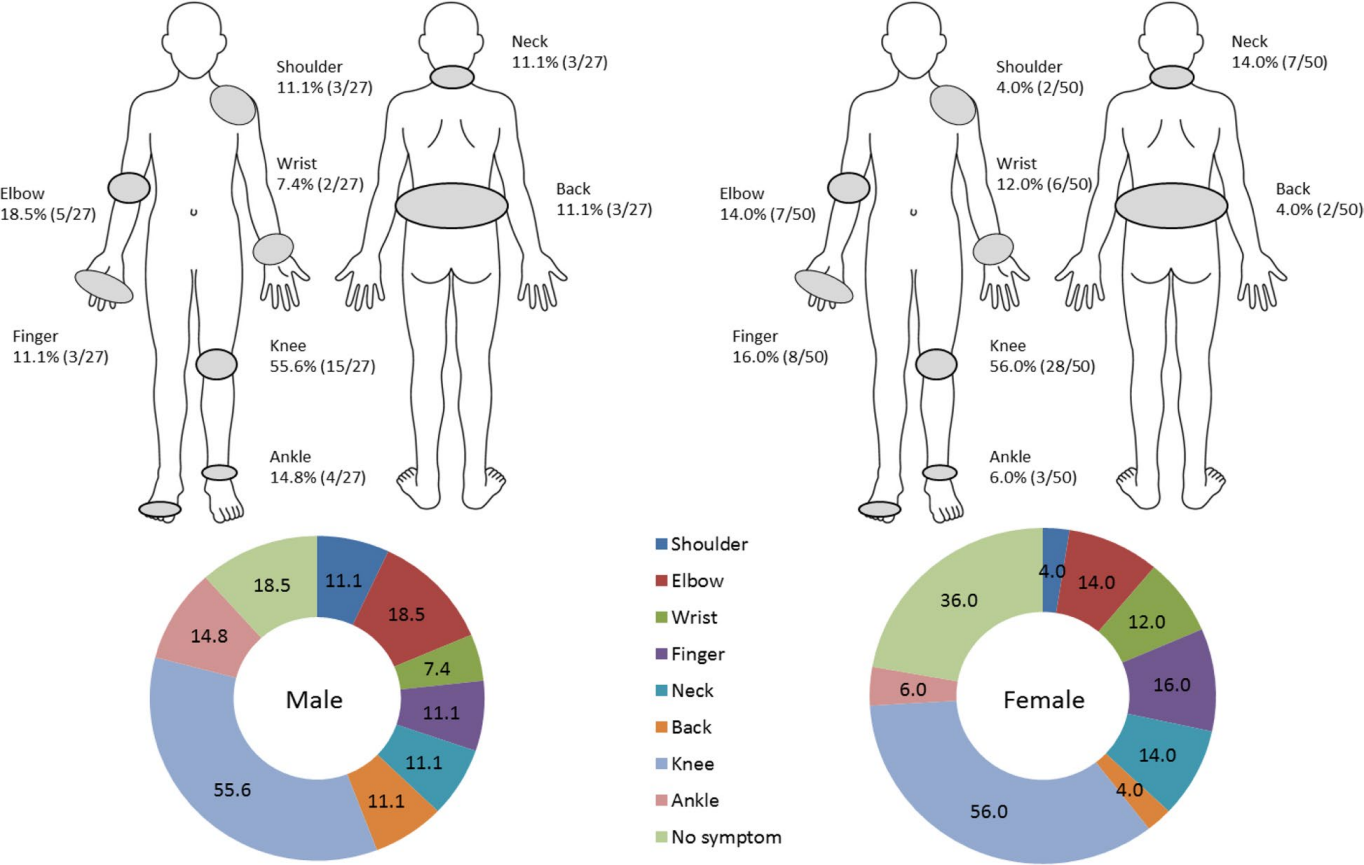
Joint sites with swelling or tenderness by sex. The prevalence of subjects who had no symptoms of arthritis was 18.5% (*n* = 5) for males and 36.0% (*n* = 18) for females. No participants complained of hip, toe, or waist swelling or tenderness. Fingers include both hands and feet

Knees (55.6%), elbows (18.5%), and ankles (18.5%) were the joints most commonly affected by arthritis in males, and knees (56.0%), fingers (16.0%), elbows, and neck (14.0%) were the joints most commonly affected by arthritis in females (Fig. 1). The prevalence of subjects who had no symptoms of arthritis was 18.5% among males and 36.0% among females. In the evaluation of arthritis using CDAI, 88.9% of males and 64.0% of females had arthritis corresponding to remission or hypoactive rheumatoid arthritis. The proportion of males with moderate or severe arthritis was 11.0%, and the proportion of females was 36.0%. Thus, a significant sex difference was observed in the degree of joint damage (*p* = 0.0304) (Table 4).

**Table 4:** Classification of strength of arthritis symptoms by the CDAI

Variables	Male (*n* = 27)		Female (*n* = 50)		*p* value
	*n*	%	*n*	%	
CDAI					
Remission/low	24	88.9	32	64.0	0.0304
Moderate/high	3	11.1	18	36.0	

Data were analyzed by Fisher’s exact tests for comparisons of variables between males and females

### Prevalence of rheumatoid arthritis by ACR/EULAR 2010 score

According to the RF measurement results, the subjects were classified as negative (male: 78.3%, female: 78.6%), weakly positive (male: 17.4%, female: 16.7%), or strongly positive (male: 4.3%, female: 4.8%). There were no significant sex differences in the proportions (*p* = 0.9940) (Table 5). The overall proportion of subjects who were positive for RF was 21.7% for males and 21.5% for females. The subjects were classified as negative (male: 100.0%, female: 95.2%) or strongly positive (male: 0.0%, female: 4.8%) according to the anti-CCP antibody level. No significant sex difference was found (*p* = 0.2878). In terms of the CRP positive rate, 30.4% of males were positive, and 21.4% of females were positive. In terms of the hyaluronic acid positive rate, 22.7% of males were positive, and 33.3% of females were positive, but neither measurement showed a significant sex difference (*p* = 0.5485, *p* = 0.5654). Furthermore, these criteria classified 4.3% of males and 7.1% of females as having rheumatoid arthritis, indicating a very high estimated prevalence.

**Table 5:** Results of biochemical tests and scores by ACR/EULAR classification for rheumatoid arthritis

Variables	Male (*n* = 23)		Female (*n* = 42)		*p* value
	*n*	%	*n*	%	
RF					
Negative	18	78.3	33	78.6	0.9940
Weakly positive	4	17.4	7	16.7	
Strongly positive	1	4.3	2	4.8	
Anti-CCP antibody					
Negative	23	100.0	40	95.2	0.2878
Weakly positive	0	0.0	0	0.0	
Strongly positive	0	0.0	2	4.8	
CRP					
Low	16	69.6	33	78.6	0.5485
High	7	30.4	9	21.4	
Hyaluronic acid^a^					
Low	17	77.3	28	66.7	0.5654
High	5	22.7	14	33.3	
ACR/EULAR 2010					
0–3	18	78.3	34	81.0	0.9943
4–5	4	17.4	5	11.9	
6≦	1	4.3	3	7.1	

Data were analyzed by Fisher’s exact tests or Cochran-Armitage trend tests for comparison of variables between males and females

Position: After the paragraph in section “Prevalence of rheumatoid arthritis by ACR/EULAR 2010 score” in the “Results” section

^a^One male participant could not be measured due to a shortage of samples

### Factors affecting ACR/EULAR 2010 score

Table 6 shows the correlation coefficient (*r*) between the ACR/EULAR2010 score and each explanatory variable, such as sociological factors and smoking habits and alcohol consumption, and the partial regression coefficient and *p* value from linear regression analysis. In males, the absolute value of the correlation coefficient between marriage status and smoking against the ACR/EULAR2020 score was over 0.2. In females, the absolute value of the correlation coefficient between age, drinking habits, and living in urban areas against the ACR/EULAR2020 score was over 0.2. In addition, in the linear regression analysis, neither factor had a significant linear relationship with the ACR/EULAR2020 score for males or females. Furthermore, multiple linear regression analysis was performed (Table 7). As a result, none of the factors were associated with ACR/EULAR 2010 scores in males. On the other hand, in females, two factors, older age (standardized parameter estimate = 4.84E−01, 95% CI = [9.19E−02, 8.76E−01], *p* = 0.0170) and history of urban residence (standardized parameter estimate = − 5.49E−01, 95% CI = [− 9.21E−01, 1.77E−01], *p* = 0.0050), significantly increased the ACR/EULAR 2010 score.

**Table 6:** Spearman’s rank correlation coefficient and linear regression analysis for ACR/EULAR2010 scores

Correlation coefficient (*r*)	ACR/EULAR2010	
	Male	Female
Age	0.1192	0.2083
Marriage status	0.2171	0.0000
Drinking habits	0.0947	0.3349
Smoking habits	− 0.2734	0.0859
Living experience in urban	0.1166	− 0.2417
Linear regression analysis (*p* value)	ACR/EULAR2010	
	Male	Female
Age	0.6080	0.1540
Marriage status	0.1410	0.8726
Drinking habits	0.5133	0.0571
Smoking habits	0.7663	0.6258
Living experience in urban	0.4878	0.1513

Analysis was performed with 23 males and 42 females who were able to provide blood. The upper half shows the correlation coefficient, and the lower half shows the *p* value for linear regression analysis

**Table 7:** Sociological factors and lifestyle habits that affect ACR/EULAR2010 scores

Variables	Male (*n* = 23)			
	Estimate	95% CI	Std.Error	*p* value
Intercept	− 1.14E−16	[− 4.43E−01, 4.43E−01]	2.10E−01	1.0000
Age	8.10E−02	[− 4.26E−01, 5.88E−01]	2.41E−01	0.7404
Marriage status	5.02E−01	[− 3.96E−02, 1.04E−00]	2.57E−01	0.0671
Drinking habits	1.90E−02	[− 4.83E−01, 5.21E−01]	2.38E−01	0.9374
Smoking habits	− 3.09E−01	[− 8.39E−01, 2.22E−01]	2.51E−01	0.2360
Living experience in urban	1.72E−01	[− 3.07E−01, 6.50E−01]	2.27E−01	0.4599
Variables	Female (*n* = 42)			
	Estimate	95% CI	Std.Error	*p* value
Intercept	3.12E−16	[− 2.83E−01, 2.83E−01]	1.39E−01	1.0000
Age	4.84E−01	[9.19E−02, 8.76E−01]	1.93E−01	0.0170
Marriage status	6.61E−02	[− 2.33E−01, 3.66E−01]	1.48E−01	0.6571
Drinking habits	1.63E−01	[− 1.50E−01, 4.76E−01]	1.54E−01	0.2974
Smoking habits	5.30E−02	[− 2.53E−01, 3.59E−01]	1.51E−01	0.7276
Living experience in urban	− 5.49E−01	[− 9.21E−01, 1.77E−01]	1.84E−01	0.0050

Analysis was performed with 23 males and 42 females who were able to provide blood samples using multiple linear regression analysis. “Estimate” in the table indicates a standardized parameter estimate, and its 95% CI is also listed

### Factors affecting CDAI score

Table 8 shows the correlation coefficient (*r*) between the CDAI score and each explanatory variable, such as sociological factors and smoking habits and alcohol consumption, and the partial regression coefficient and *p* value by linear regression analysis. In males, the absolute value of the correlation coefficient between age, marriage status, drinking habits, and smoking habits against the CDAI score was over 0.2. In females, the absolute value of the correlation coefficient between age, marital status, and smoking habits against the CDAI score was over 0.2. In addition, in the linear regression analysis, marriage status, and smoking habits had a significant linear relationship with the CDAI score in females. Furthermore, multiple linear regression analysis was performed (Table 9). As a result, none of the factors were associated with CDAI scores in males. On the other hand, in females, three factors, having no spouse (standardized parameter estimate = 3.17E−01, 95% CI = [5.74E−02, 5.77E−01], *p* = 0.0179), smoking habits (standardized parameter estimate = 2.88E−01, 95% CI = [1.71E−02, 5.59E−01], *p* = 0.0377), and a history of urban residence (standardized parameter estimate = − 3.69E−01, 95% CI = [− 6.83E−01, − 5.60E−02], *p* = 0.0219), significantly increased CDAI scores.

**Table 8:** Spearman’s rank correlation coefficient and linear regression analysis for CDAI scores

Correlation coefficient (*r*)	CDAI	
	Male	Female
Age	0.2842	0.2151
Marriage status	0.3103	0.2067
Drinking habits	0.3648	0.0463
Smoking habits	0.2300	0.2056
Living experience in urban	0.0740	− 0.1964
Linear regression analysis (*p* value)	CDAI	
	Male	Female
Age	0.6970	0.2650
Marriage status	0.2780	0.0490
Drinking habits	0.3540	0.5744
Smoking habits	0.5817	0.0422
Living experience in urban	0.8030	0.2318

Analysis was performed with 27 males and 50 females who were able to provide blood. The upper half shows the correlation coefficient, and the lower half shows the *p* value for linear regression analysis

**Table 9:** Sociological factors and lifestyle habits that affect CDAI scores

Variables	Male (*n* = 27)			
	Estimate	95% CI	Std.Error	*p* value
Intercept	5.30E−18	[− 4.28E−01, 4.28E−01]	2.06E−01	1.0000
Age	3.87E−02	[− 4.56E−01, 5.34E−01]	2.38E−01	0.8720
Marriage status	1.93E−01	[− 3.27E−01, 7.13E−01]	2.50E−01	0.4480
Drinking habits	1.58E−01	[− 3.35E−01, 6.50E−01]	2.37E−01	0.5130
Smoking habits	3.48E−03	[− 5.06E−01, 5.13E−01]	2.45E−01	0.9890
Living experience in urban	− 6.13E−02	[− 5.13E−01, 3.90E−01]	2.17E−01	0.7810
Variables	Female (*n* = 50)			
	Estimate	95% CI	Std.Error	*p* value
Intercept	1.15E−17	[− 2.55E−01, 2.55E−01]	1.27E−01	1.0000
Age	3.05E−01	[− 3.49E−02, 6.45E−01]	1.69E−01	0.0774
Marriage status	3.17E−01	[5.74E−02, 5.77E−01]	1.29E−01	0.0179
Drinking habits	− 7.45E−02	[− 3.55E−01, 2.06E−01]	1.39E−01	0.5954
Smoking habits	2.88E−01	[1.71E−02, 5.59E−01]	1.34E−01	0.0377
Living experience in urban	− 3.69E−01	[− 6.83E−01, − 5.60E−02]	1.56E−01	0.0219

Analysis was performed with 27 males and 50 females by multiple linear regression analysis. “Estimate” in the table indicates a standardized parameter estimate, and its 95% CI is also listed

### Effects of smoking habits on joint symptoms

Table 10 shows the correlation coefficient (*r*) between smoking habits and each explanatory variable, such as activity limitation, joint disability, and serum data, and the partial regression coefficient and *p* value by linear regression analysis. In males, the absolute values of the correlation coefficients between large joint disability, CRP, and hyaluronic acid against smoking habits were over 0.2. In females, the absolute value of the correlation coefficient between CRP and hyaluronic acid was over 0.2. In addition, in the linear regression analysis, only hyaluronic acid had a significant linear relationship with smoking habits in males. Furthermore, multiple linear regression analysis was performed (Table 11). As a result, hyaluronic acid was significantly higher in both males (standardized parameter estimate = 6.03E−01, 95% CI = [3.06E−01, 9.01E−01], *p* = 0.0020) and females (standardized parameter estimate = 4.87E−01, 95% CI = [5.63E−02, 9.18E−01], *p* = 0.0291) who had a smoking habit.

**Table 10:** Spearman’s rank correlation coefficient and linear regression analysis for smoking habits.

Correlation coefficient (*r*)	Smoking	
	Male	Female
Activity limitation	− 0.1371	− 0.0953
Small joint disability	0.1643	0.0310
Large joint disability	0.3323	0.1972
Rheumatoid factor	0.1149	0.1149
CRP	− 0.2717	− 0.2717
Hyaluronic acid	0.2297	0.2297
Linear regression analysis (*p* value)	Smoking	
	Male	Female
Activity limitation	0.8119	0.5323
Small joint disability	0.5320	0.7570
Large joint disability	0.5140	0.1810
Rheumatoid factor	0.2920	0.4620
CRP	0.1730	0.2560
Hyaluronic acid	0.0086	0.3370

Analysis was performed with 23 males and 42 females who were able to provide blood. The upper half shows the correlation coefficient, and the lower half shows the *p* value for linear regression analysis

**Table 11:** Effects of smoking on joint disorders and serological test results

Variables	Male (*n* = 23)			
	Estimate	95% CI	Std.Error	*p* value
Intercept	− 3.54E−01	[− 5.96E−01, − 1.11E−01]	1.03E−01	0.0108
Activity limitation	1.56E−01	[− 1.81E−01, 4.92E−01]	1.42E−01	0.3101
Small joint disability	3.33E−03	[− 2.24E−01, 2.30E−01]	9.60E−02	0.9733
Large joint disability	2.02E−02	[− 2.74E−01, 3.14E−01]	1.24E−01	0.8756
Rheumatoid factor	7.71E−03	[− 2.58E−01, 2.74E−01]	1.12E−01	0.9472
CRP	4.25E−03	[− 2.40E−01, 2.49E−01]	1.03E−01	0.9684
Hyaluronic acid	6.03E−01	[3.06E−01, 9.01E−01]	1.26E−01	0.0020
Variables	Female (*n* = 42)			
	Estimate	95% CI	Std.Error	*p* value
Intercept	− 1.54E−01	[− 5.53E−01, 2.46E−01]	1.88E−01	0.4269
Activity limitation	− 2.52E−01	[− 7.09E−01, 2.05E−01]	2.16E−01	0.2598
Small joint disability	3.70E−01	[− 1.35E−00, 2.09E−00]	8.13E−01	0.655
Large joint disability	− 1.04E−01	[− 6.99E−01, 4.92E−01]	2.81E−01	0.7161
Rheumatoid factor	− 4.04E−01	[− 1.83E−00, 1.02E−00]	6.74E−01	0.5572
CRP	− 3.54E−01	[− 5.96E−01, − 1.11E−01]	1.03E−01	0.0108
Hyaluronic acid	1.56E−01	[− 1.81E−01, 4.92E−01]	1.42E−01	0.3101

Analysis was performed with 23 males and 42 females who were able to provide blood samples using multiple linear regression analysis. “Estimate” in the table indicates a standardized parameter estimate, and its 95% CI is also listed

Position: After the paragraph in section “Effects of smoking habits on joint symptoms” in the “Results” section (after Table 10)

## Discussion

### Characteristics of Tsarang residents

In terms of the use of alcohol and tobacco among the subjects, the alcohol consumption history of males was significantly higher than that of females, but there was no difference between males and females in smoking history. A survey of 2815 adults in the Pokhara district in 2013 found that 67.2% of males and 18.9% of females had consumed alcohol [35]. According to this study, the alcohol consumption rate of Tsarang residents is 81.5% for males and 42.0% for females, which may be higher than that in urban areas. In addition, the smoking rate in Nepal decreases annually, from 41% in 2000 to 35.5% in 2015 for males and from 28% in 2000 to 9% in 2015 for females [36]. In this study, 40.0% of females had a history of smoking, and highland females may have more opportunities to come into contact with tobacco than females in other regions in this country. Thus, it was suggested that females living in Mustang may have higher smoking and alcohol consumption rates than those living in urban areas. Smoking and drinking habits may be a public health problem, especially among high-altitude Mustang residents, because smoking habits have been reported to lead to vascular aging, reduced SpO_2_, and risk factors for rheumatoid arthritis [37–39].

### Comparison of arthritis prevalence with other regions

In an American survey conducted between 2013 and 2015, 18.1% of males and 23.5% of females suffered from arthritis [40]. In a survey of arthritis in low- to middle-income countries, Russia had the highest rate of arthritis in the past year: 32.9% among males and 48.4% among females [41]. Comparison with these previous studies suggests that the high prevalence of arthritis in the Tsarang population may be a major public health problem.

Although no significant difference was observed between males and females in this study, the results suggest that females may be more strongly affected by arthritis in their daily lives. In fact, the proportion of people who went to the clinic or medical facility for arthritis and took medication was significantly higher among females than among males. Previous reports have shown that rheumatoid arthritis and osteoarthritis are more prevalent in females, and even infectious psoriatic arthritis is more likely to cause pain and dysfunction in females [42–44].

In this study, the knees were the most commonly impaired joints in both males and females. Upper Mustang is an area at one of the highest altitudes in the Mustang area. Many residents live in this area while farming or handling livestock, accounting for 45.2% of the total population in that region [45]. Since Tsarang, the study site, is located in Upper Mustang, it is considered that these types of physical labor place a burden on the knees. The next most common site of disability was the elbow in males and the finger in females. Males are more likely to be engaged in manual labor, and elbows and knees may be impaired. On the other hand, the proportion of females who had swelling or pain in the small joints of the fingers, which is a characteristic of rheumatoid arthritis, was higher than that of males. Therefore, it can be said that there was a sex difference in the characteristics of arthritis among Tsarang residents. Furthermore, in this study, the degree of disability associated with arthritis was evaluated using the CDAI. The CDAI is an index that was originally developed to evaluate the disease activity of patients diagnosed with rheumatoid arthritis, but in this study, the residents’ CDAI score was calculated to widely evaluate the presence or absence of arthritis and its severity among all participants, not limited to patients with rheumatoid arthritis. As a result, the rate of arthritis corresponding to moderate or high rheumatoid arthritis activity was significantly higher in females than in males.

Serological tests were conducted for rheumatoid factor, which increases in 50–80% of patients with rheumatoid arthritis, anti-CCP antibody, which has 98% specificity for rheumatoid arthritis diagnosis [46], CRP, which reflects a systemic inflammatory response, and hyaluronic acid, which increases in people who have experienced joint destruction [47]. A study of non-arthritic groups in Finland found an RF-positive rate of 2.1% and a strong positive rate of 1.0% [48]. In addition, a 2013 survey of the general population in Turkey found that 2.8% were RF-positive and 1% were anti-CCP antibody-positive [46]. Although these previous studies included data from younger people than this study because they targeted people aged 18 years and older, it was found that the positive rate of RF and anti-CCP antibodies in Tsarang residents was high.

### Prevalence of rheumatoid arthritis

The standard currently used worldwide is the ACR/EULAR 2010, which was jointly created by the Rheumatoid Arthritis Society of the USA and Europe [34]. Many studies evaluating the prevalence of rheumatoid arthritis have used the 1987 or 2020 criteria, and comparing the prevalence of rheumatoid arthritis between such studies should be done carefully. However, even considering these differences in classification criteria, it was clear that the prevalence of rheumatoid arthritis in the Tsarang population obtained in this study was high. In a study that evaluated rheumatoid arthritis in Tibetan highlanders in Naku, China, using ACR/EULAR 2010, as in this study, it was reported that 4.86% of the subjects may have rheumatoid arthritis [49]. Studies comparing ancient human bone DNA with modern human DNA in the Annapurna Conservation Area, to which Mustang belongs, have previously reported that ancient human genes are still strongly inherited by the Mustang people [50]. In other words, the people of Mustang may strongly inherit the hypoxic adaptation genes of ancient Tibetan highlanders. Therefore, if the hypoxic adaptation mechanism of Tibetan highlanders promotes the development of rheumatoid arthritis through the expression of HIF, the prevalence of rheumatoid arthritis may be especially high in Tibetan highlanders living in Mustang. The relationship between the hypoxic adaptation gene and the onset of rheumatoid arthritis in Tibetan highlanders needs further verification from a genetic perspective.

### Risk factors for rheumatoid arthritis among Tibetan highlanders

Previous studies have reported risk factors for rheumatoid arthritis, such as sex, age, heredity, smoking, exposure to particles of asbestos and silica, and obesity [20, 51]. In this study, we explored risk factors for rheumatoid arthritis in Tsarang residents by conducting linear multiple regression analysis using the ACR/EULAR 2010 score, which is a classification standard for rheumatoid arthritis, as the objective variable. It is not clear in this study how migrating to work in urban areas (lowlands) is a risk factor for rheumatoid arthritis. One possibility is that if highlanders born and raised in the highlands descend to the lowlands and then return to the highlands, the body will try to adapt to hypoxia again, and the expression level of HIF will temporarily increase. If so, highlanders who have lived in urban areas may be at increased risk of rheumatoid arthritis through overexpression of HIF.

Multiple regression analysis revealed that smoking, which has been reported as a risk factor for rheumatoid arthritis, may be widely involved in the development of arthritis, even at high altitudes. Furthermore, according to the results obtained by analyzing the relationship between the smoking habits of residents and joint disorders and biochemical factors in the blood, individuals with smoking habits tended to have higher blood hyaluronic acid levels among both males and females. Smoking was found to be a risk factor for arthritis in Tsarang residents.

The synovium is a loose connective tissue that constitutes the joint capsule. Synovial cells exist in the surface layer, and blood vessels and lymph vessels exist in the deep layer [52]. Therefore, the synovial fluid produced by the synovium is supplied to the joint cavity, and nutrients and oxygen are delivered to the cartilage where blood vessels are not distributed [52]. Inflammation of the synovium is considered to be one of the major pathologies of rheumatoid arthritis and results in severe tissue edema and overgrowth of approximately 10–15 layers of synovial cells [53]. In patients with rheumatoid arthritis, the expression of hypoxia-inducible factor (HIF) is elevated in fibroblast-like synovial cells (FLSs) and macrophage-like synovial cells (MLSs), which make up the inflamed hyperplastic synovium [54, 55]. In particular, FLSs, along with inflammatory cells, have been reported to be strongly involved in the onset and exacerbation of rheumatoid arthritis [56]. HIF-α has three isoforms (HIF-1α, HIF-2α, HIF-3α), and it has been reported that HIF-2α is mainly expressed in FLSs in the joint cavity of patients with rheumatoid arthritis [25, 57]. Therefore, smoking habits in the Tibetan Plateau may promote HIF production in the synovium and promote the development of rheumatoid arthritis. Further molecular biological verification is needed in the future.

Given that the primary purpose of evolution and adaptation is to leaving offspring, diseases such as rheumatoid arthritis, which often develop after the age of reproduction, have little effect on reproduction. Even if such disadvantages are inherent, it is considered that the selection that prioritizes adaptation to a hypoxic environment has been applied. It is possible that vulnerability against diseases such as rheumatoid arthritis have become apparent due to the increase in life expectancy and the introduction of tobacco among Tibetan highlanders.

### Limitations of this study

In this study, we surveyed the inhabitants of Tsarang in the less populated Charang area. According to 2011 data, the population of the Charang region is reported to be 119 males and 131 females over the age of 35 [29]. No population data have been reported since then, but there have been no major fluctuations, and the population is still considered to be approximately the same. Although the representativeness in statistical analysis is not high, data and samples from the 1930s to the 1980s were acquired and analyzed. Having analyzed the characteristics of Tsarang populations, further research is needed to clarify their association with rheumatoid arthritis prevalence and hypoxic adaptation in Tibetan highlanders throughout Mustang.

## Conclusions

In this study, we evaluated the symptoms of arthritis and estimated the prevalence of rheumatoid arthritis using classification criteria for Tibetan highlanders who have adapted to the hypoxic environment and fostered their own culture. The high prevalence of rheumatoid arthritis among Tsarang residents suggests that the hypoxic adaptation mechanism involving HIF in Tibetan highlanders may promote the onset or exacerbation of rheumatoid arthritis. The results of this study are valuable not only because of the anthropological characteristics of Tibetan highlanders but also because of the geographical isolation of Tsarang from urban areas. In addition, valuable results were obtained for both clinical patients with rheumatoid arthritis and the general population. In addition, this study is the first report describing the site of joint damage and its effect on daily life among Tibetan highlanders in detail. The high prevalence of rheumatoid arthritis among Tibetan highlanders may be related not only to the environmental factors analyzed in this study but also to hypoxic adaptation genes. Further investigation is needed to clarify the genetic factors involved.

## Data Availability

The datasets generated during the current study are not publicly available, but are available from the corresponding author on reasonable request.

## Abbreviations

BMI: Body mass index
CCP: Cyclic citrullinated peptide
CDAI: Clinical disease activity index
CRP: C-reactive protein
EPAS1: Endothelial per-ARNT-sim domain protein 1
FLSs: Fibroblast-like synovial cells
HIF: Hypoxia-inducible factor
MLSs: Macrophage-like synovial cells
SD: Standard deviation
SpO_2_: Saturation of peripheral oxygen
